# From Signals to Measurands: A Measurement-Science Roadmap for Reproducible Analytical Biochemistry

**DOI:** 10.1021/acsmeasuresciau.6c00198

**Published:** 2026-09-07

**Authors:** Santiago Schnell

**Affiliations:** † Department of Mathematics, 3728 Dartmouth College, Hanover, New Hampshire 03755, United States; ‡ Departments of Biochemistry & Cell Biology and Biomedical Data Science, Geisel School of Medicine at Dartmouth, Hanover, New Hampshire 03755, United States

**Keywords:** measurement science, measurand, enzyme kinetics, ligand binding, measurement uncertainty, identifiability, reproducibility

## Abstract

Analytical biochemistry often reports kinetic or binding constants as though they were observed directly. Each result is instead inferred from an instrumental signal. Calibration establishes the scale, biochemical and observation models connect the signal to a target, and statistical analysis produces the estimate and its uncertainty. This Perspective treats reproducibility as the recovery of a specified measurand, with an uncertainty statement, through an auditable measurement chain. A Michaelis–Menten substrate-depletion example provides the organizing case. Two progress-curve experiments generated with the same parameters and noise model both fit well, yet only the design that samples substrate concentrations around the Michaelis constant yields a bounded interval for *K*_M_. In that design, uncertainty in active-site concentration dominates the uncertainty of *k*_cat_ even though the curve fit is precise. Ligand–receptor binding shows parallel effects of depletion, signal modeling, and parameter confounding. The framework connects established metrological and chemical-measurement concepts with enzymology reporting standards, machine-readable data formats, and modern identifiability analysis. It distinguishes repeatability, transfer to another laboratory, and agreement across independent measurement routes (or techniques), and explains what interlaboratory studies can reveal. The recommendations are prioritized as minimum practice, stronger support for parameter estimation, and reference-grade validation. The same logic applies whenever a biochemical quantity is inferred from a measured signal.

## Graphical abstract

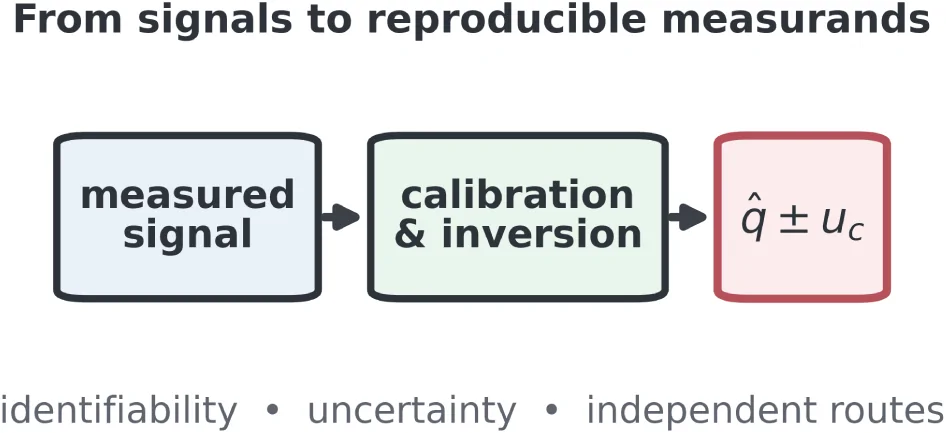

## Reproducibility Begins with a Measurement Question

A familiar problem in analytical biochemistry appears simple. A laboratory attempts to recover a published Michaelis constant, *K*_M_, for a well-studied enzyme. The protocol is followed carefully, the time courses are clean, and repeated runs give a stable estimate. Yet that estimate differs from the published value by a factor of two. Before asking which laboratory is wrong, one must ask whether the two numbers refer to the same biochemical quantity and whether either experiment constrained it.

An absorbance or binding trace does not arrive labeled *K*_M_, *k*_cat_, or *K*_D_. The reported quantity emerges only after the signal has been calibrated, related to a biochemical model, analyzed statistically, and assigned an uncertainty. The numerical result is therefore an inference from a measured signal to a specified measurand—the quantity intended to be measured under stated conditions. What the instrument records and what the constant denotes are distinct objects: the trace is data, whereas the constant is a phenomenon inferred from it.^1–5^

This Perspective is addressed primarily to experimental analytical biochemists who infer quantitative results from instrumental signals. It should also be useful to measurement scientists and quantitative analysts concerned with comparison across experiments and laboratories. No prior training in metrology or identifiability analysis is assumed.

Three problems are often grouped under the word reproducibility. An experiment can fail in execution because it was not performed as described. A report can fail in description because material information or tacit procedural knowledge is missing. A measurement can also fail in inference: the work was competently performed, yet the design or model did not support the claimed quantity. Reporting studies document the first two problems, whereas identifiability studies show why the third can persist even when a fitted curve looks satisfactory.^6–10^ These three failures call for different evidence, and the word *reproducibility* obscures the difference. This Perspective therefore separates three checks that probe progressively more of the measurement: *repeatability*, *transfer replication*, and *independent-method reproducibility*. They are defined in the section *From repeatability to interlaboratory evidence* and summarized in Table 3.

The central claim is that reproducibility concerns the compatible recovery of a specified measurand, with uncertainty, after the experimental and inferential conditions have been made explicit. The contribution is integrative. It joins metrological definitions to biochemical model validity, parameter identifiability, uncertainty evaluation, and reusable computational records.^11–13^

## A Michaelis–Menten Substrate-Depletion Case Study

To make the problem concrete, consider the single-enzyme, single-substrate Michaelis–Menten reaction mechanism with irreversible product formation,

1 $$E+S\underset{k_{-1}}{\overset{k_{1}}{⇌}}\mathrm{ES}\overset{k_{\mathrm{cat}}}{\to }E+P$$

For this reaction scheme,

2 $$K_{S}=\frac{k_{-1}}{k_{1}},,K=\frac{k_{\mathrm{cat}}}{k_{1}},,K_{M}=\frac{k_{-1}+k_{\mathrm{cat}}}{k_{1}}=K_{S}+K$$

Here *K*_S_ is the dissociation constant of the enzyme–substrate complex, *K* is the Van Slyke–Cullen constant, and *K*_M_ is the Michaelis constant. The Michaelis constant is not generally an equilibrium dissociation constant. It approaches *K*_S_ only in the rapid-equilibrium, or slow-catalysis, limit *k*_cat_ ≪ *k*_–1_.^14–16^

Under the usual quasi-steady-state reduction, with negligible substrate loss during the initial transient, substrate depletion is described by

3 $$\frac{d\left[S\right]}{dt}=-\frac{V\left[S\right]}{K_{M}+\left[S\right]},,V=k_{\mathrm{cat}}{[E]}_{0}$$

where [E]_0_ is the initial concentration of catalytically active enzyme sites. The corresponding progress curve is

4 $$\left[S\right]\left(t\right)=K_{M}W_{0}\left[\frac{{[S]}_{0}}{K_{M}}\exp\left(\frac{{[S]}_{0}-Vt}{K_{M}}\right)\right]$$

with *W*_0_ denoting the principal real branch of the Lambert *W* function. This is the Schnell–Mendoza equation.^17–20^

In the worked example, substrate depletion is followed spectrophotometrically through A=εl[S] . Both experiments use *K*_M_ = 50 μM, *V* = 0.20 μM s^–1^, the same absorption coefficient and path length, and the same additive absorbance-noise model. The initial substrate concentration is treated as a separately calibrated input obtained from an independent initial absorbance measurement on the same εl scale; it is not estimated from the first noisy observation in the progress curve. Repeatability uncertainty in that separate initial measurement and uncertainty in the 1 cm path length are neglected in this illustrative in silico example. Design I begins at [S]_0_ = 10 μM (0.2 *K*_M_), so the measured progress curve remains entirely below the Michaelis constant. Design II begins at [S]_0_ = 200 μM (4 *K*_M_) and follows substrate depletion through concentrations on both sides of *K*_M_. Because the two designs deplete substrate on different time scales, Design I is sampled at 50 points over 1000 s and Design II at 60 points over 1800 s, each window spanning a comparable extent of depletion. Figure 1A shows the measured substrate concentrations and the fitted progress curves. Three Design I concentration estimates are nonpositive because the additive absorbance noise exceeds the residual signal near depletion. They remain in the fit and are shown as downward triangles at a declared plotting floor. Both fits have reduced *χ* ^2^ values close to one.

**Figure 1:**
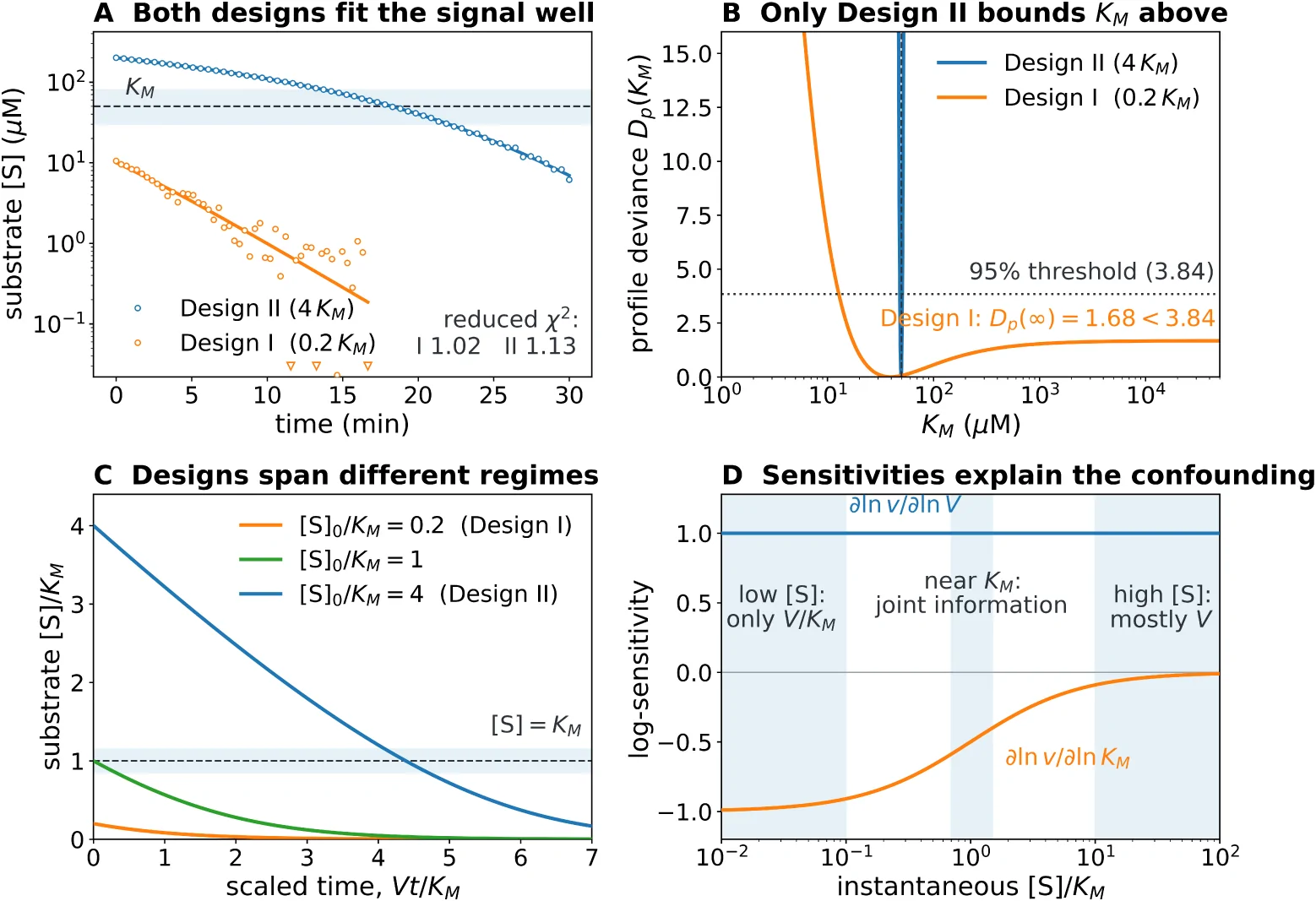
Substrate-depletion progress curves under different Michaelis–Menten experimental designs. **A** Designs I and II use the same biochemical parameters and absorbance-noise model, and both fitted curves describe the measured signal well. Three nonpositive Design I concentration estimates are retained in the fit and shown by downward triangles at the plotting floor because the ordinate is logarithmic. **B** The profile deviance *D_p_* (*K*_M_) gives a bounded nominal 95% interval only for Design II; for Design I, the limiting value *D_p_* (∞) = 1.68 remains below the 3.84 threshold, so no finite upper bound exists, although both designs constrain the combination *V*/*K*_M_. **C** Dimensionless progress curves beginning below, at, and above the Michaelis constant, the first and last corresponding to Designs I and II, show which substrate regimes are traversed. **D** Local log sensitivities explain why low substrate chiefly determines *V*/*K*_M_, high substrate chiefly determines *V*, and concentrations near *K*_M_ help separate the parameters.

The difference appears when *K*_M_ is challenged directly. For each trial value of *K*_M_, *V* is re-estimated and the best conditional fit is compared with the overall best fit. The loss of fit is summarized by the profile deviance

5 $$D_{p}\left(K_{M}\right)=-2\left\{l_{p}\left(K_{M}\right)-l_{\max}\right\}$$

where $l_{p}\left(K_{M}\right)$ is the maximized log-likelihood at fixed *K*_M_. Under the usual likelihood-ratio regularity conditions, *D*_*p*_ = 3.84 is the nominal 95% threshold for one profiled parameter. Design II crosses that threshold on both sides of its optimum and gives a finite interval. Design I has no finite upper bound (Figure 1B). This conclusion is not inferred solely from the finite profiling grid: in the limiting model [S]­(*t*) = [S]_0_exp­[−(*V*/*K*_M_)*t*] as *K*_M_ → ∞, re-estimation of *V*/*K*_M_ gives *D*_*p*_(∞) = 1.68, below the nominal threshold.^8,9^

The reason can be seen directly from the rate law. When [S] ≪ *K*_M_ throughout the observation window,

6 $$\frac{d\left[S\right]}{dt}\approx -\frac{V}{K_{M}}\left[S\right]$$

The substrate-depletion progress curve is then essentially exponential and determines the combination *V*/*K*_M_. At high substrate, the rate is close to *V* and carries little information about *K*_M_. Separate estimation requires observations near [*S*] ∼ *K*_M_, where the two parameters affect the curve differently. Figure 1C shows substrate-depletion progress curves that begin in these different regimes, and Figure 1D shows the corresponding local sensitivities.^16,21^

Simulation-and-recovery confirms the profile diagnosis. Across 400 synthetic data sets per design, the central 95% of recovered *K*_M_ values extends from about 15 μM to the 10 mM search limit in Design I, whereas Design II recovers 48.6–51.3 μM. The point estimate for Design II is 49.5 μM, with a nominal 95% profile interval of 48.4–50.5 μM. The failure of Design I is a property of the substrate regime rather than of sampling effort. Given Design II’s schedule, or four times the observations over the same window, or six times the observations over five times the window, the profile region for *K*_M_ under Design I still spans between two and three orders of magnitude and remains open at the 10 mM limit of the profiled range in most replicates; the Supporting Information reports that comparison.

The point estimates themselves illustrate the same difficulty. Because *V* and *K*_M_ have different units and poorly matched numerical scales in the chosen parameterization, the fit is performed in log *V* and log *K*_M_ from several starting points. The reported reference is the lowest residual sum of squares found by that bounded multistart procedure. For Design II this choice is immaterial. For Design I it is not: a fit in the natural parameters stops about 0.05% above the best-fit numerical minimum, but at a value of KM^ some 13% away, while *V*/*K*_M_ shifts by under 2%. When a point estimate is that sensitive to optimizer tolerance and the identified combination is not, the design, rather than the software, is what needs attention.

A bounded kinetic parameter still requires a complete uncertainty evaluation. In Design II, the curve-fit relative standard uncertainty of *V* is 0.4%. Converting *V* to *k*_cat_ = *V*/[E]_0_ introduces the independently measured active-site concentration ([E]_0_ = 4.0 nM, established by titration of a nominal total of 5.0 nM), whose relative standard uncertainty is 8%, as well as a 2% absorbance-scale contribution. The combined standard uncertainty of *k*_cat_ is therefore 8.3%. The absorption coefficient affects *K*_M_ and *k*_cat_ but cancels from *k*_cat_/*K*_M_. The full likelihood, recovery study, and uncertainty budget—the itemized list of the individual contributions to the uncertainty of a result, each with its magnitude and how it was evaluated—are reported in the Supporting Information.

A concise measurand declaration for Design II is set out in Figure 5, in Prioritized Recommendations for Routine Analytical Biochemistry; the complete computational and uncertainty record is in the Supporting Information.

Table 1 sets the three contributions against the three reported quantities. The same fitted curve underlies all three results, yet the dominant source of uncertainty differs in each: calibration for *K*_M_, and active-site concentration for both *k*_cat_ and *k*_cat_/*K*_M_. Because the progress-curve observations and the separately calibrated initial substrate concentration share one absorbance scale, the absorption coefficient rescales both together and cancels from the specificity constant. That removes one calibration contribution; it does not by itself make *k*_cat_/*K*_M_ transferable, since active-site concentration, model validity, temperature, pH, and material state all remain.

**Table 1:** Relative Standard Uncertainty Budget for Design II^a^

Quantity	Type A (fit)	*u*(*ε*)/*ε*	*u*([E]_0_)/[E]_0_	*u*_*c*_	Dominant source
*K*_M_	1.3%	2.0%	—	2.4%	absorbance-scale calibration
*k*_cat_	0.4%	2.0%	8.0%	8.3%	active-site concentration
*k*_cat_/*K*_M_	0.9%	—	8.0%	8.1%	active-site concentration

a Type A components are standard uncertainties obtained from the fitted covariance; the remaining components are evaluated from calibration and material characterization. u_c_ is the combined standard uncertainty. The profile regions reported earlier are interval diagnostics of identifiability and are not combined here.

This case establishes two requirements that recur throughout the paper. The data must place useful bounds on the intended parameter, and the final uncertainty must include important inputs outside the curve fit.

## From Signal to Measurand

The Michaelis–Menten example can be generalized by distinguishing the intended quantity from the signal that is observed. A model connects them, and an analysis procedure produces the reported result. Figure 2 defines the terms used for that separation. The definitions follow the International Vocabulary of Metrology (VIM) and the Guide to the Expression of Uncertainty in Measurement (GUM), with the technical report of the International Union of Pure and Applied Chemistry (IUPAC) providing a chemical interpretation.^1–3,5^

**Figure 2:**
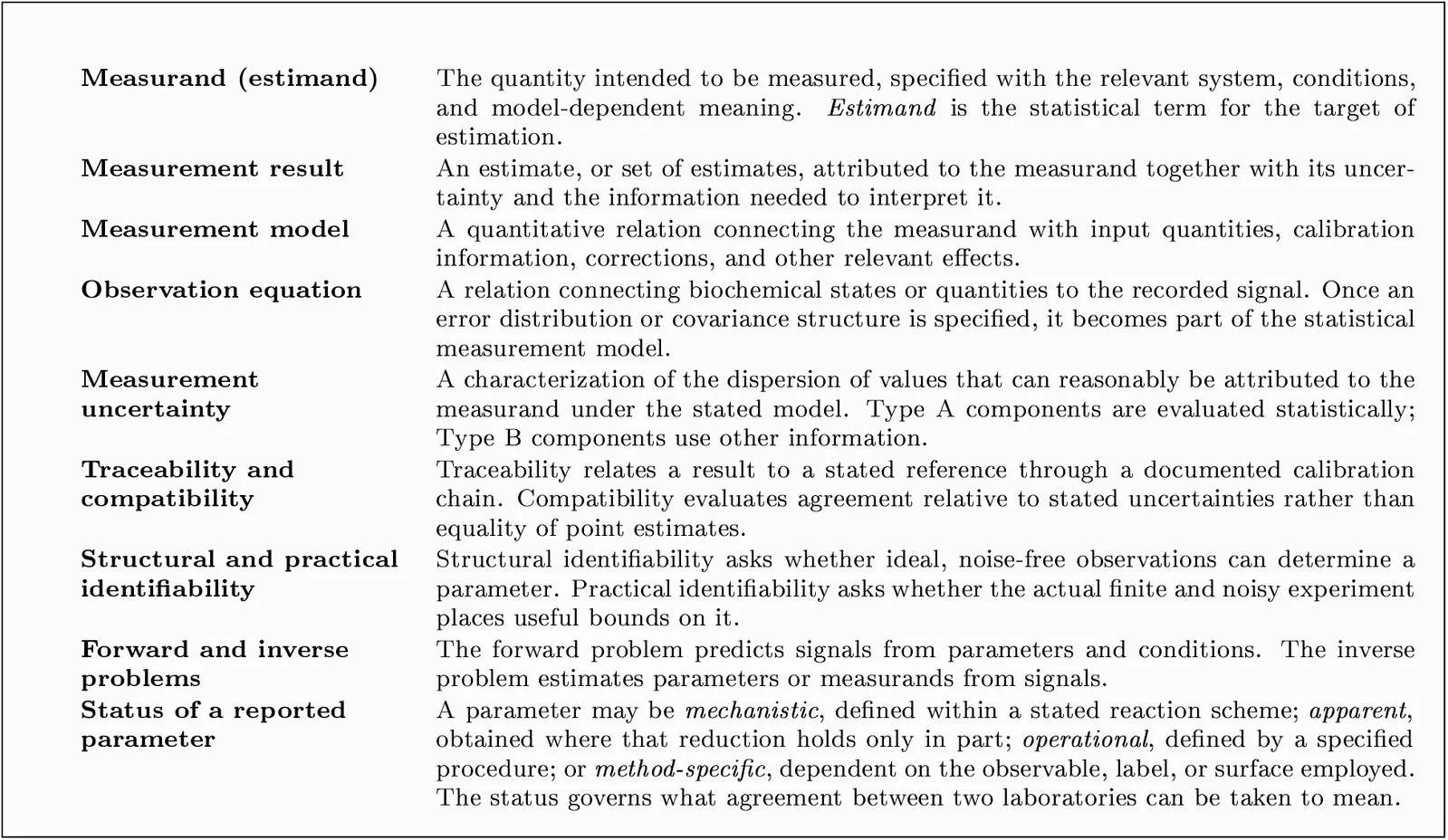
Working vocabulary for the signal-to-measurand framework.

A useful first relation is

7 q=h(θ,C)

where *q* is the intended biochemical quantity, *θ* contains the parameters that define it, and C specifies the biochemical context. eq 7 is a *measurand-defining relation*; it states what the quantity means but is not a complete measurement model. The context includes the molecular system and the conditions that can alter its behavior.

For method *m*, let *Y*_*m*_(*t*_*i*_) denote the recorded signal. An observation equation can be written schematically as

8 $$Y_{m}\left(t_{i}\right)=g_{m}\left(x\left(t_{i};\theta \right),\eta_{m},C_{m}\right)+\varepsilon_{m}\left(t_{i}\right)$$

where *x*(*t*_*i*_; *θ*) is the biochemical state, *η*_*m*_ contains method-specific nuisance parameters, and *C*_*m*_ contains calibration information. A specified distribution or covariance structure for *ε*_*m*_, together with the biochemical model for *x*, turns eq 8 into a statistical measurement model in the broad GUM-6 sense. The analysis procedure produces

9 $${\mathrm{q̂}}_{m}=A_{m}\left(Y_{m},C_{m},M_{\mathrm{bio}},M_{\mathrm{stat}}\right)$$

and the measurement result is reported as an estimate together with an uncertainty statement. The combined standard uncertainty *u*_c_(*q̂*_*m*_) collects the contributions from every input; where an expanded uncertainty is wanted, *U* = *k u*_c_, and the coverage factor *k* and the intended coverage probability must be stated alongside it. The worked results below are reported as *q̂* ± *u*_c_(*q̂*).

This hierarchy replaces the vague instruction to “fit the curve” with a sequence of explicit decisions. The investigator defines the target, states how the biochemical system produces the signal, specifies how variation is modeled, and then propagates the relevant sources of uncertainty. Table 2 summarizes the minimum record needed to make that sequence auditable.

**Table 2:** Minimum Specification along the Signal-to-Measurand Chain

Part of measurement	What the report should state	Why it matters
Material and conditions	Identify the molecular construct and the fraction capable of reaction. State the chemical environment and any label or surface that can perturb the system	Defines which biochemical system was measured and the conditions under which the result has meaning
Signal and calibration	Preserve the raw signal and describe its reference, calibration, blank correction, and instrumental limits	Establishes the scale that connects instrument response to biochemical quantity
Biochemical and observation models	State the reaction or binding model, its validity regime, and the equation that maps biochemical state to signal	Defines the parameter and prevents a method-specific descriptor from being mistaken for a more general constant
Statistical analysis	Describe the error structure, estimator, weighting, parameter bounds, and diagnostic checks	Determines which estimates and intervals are supported by the data
Result and provenance	Report the estimate with its uncertainty, limitations,software, and location of the data and code	Makes comparison, reanalysis, and transfer possible

Figure 3 groups the same stages graphically. A protocol specifies the operations, whereas the measurement and observation models specify how the result is inferred. Repeating the protocol estimates variation conditional on the entire chain; it does not establish by itself that the chain reaches the intended measurand.

**Figure 3:**
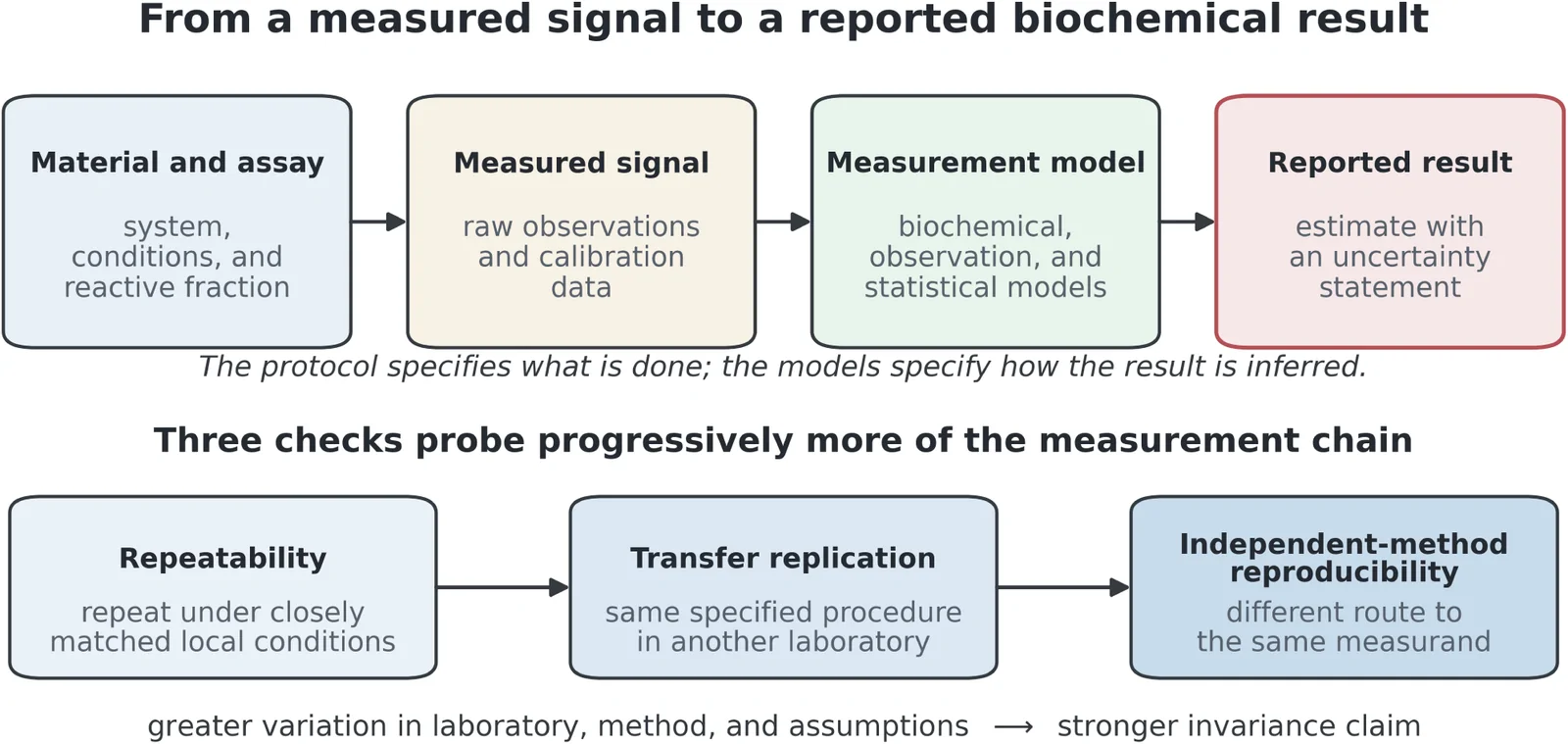
Signal-to-measurand chain and three progressively stronger checks. The upper row separates the material and assay, measured signal, measurement model, and reported result. The lower row distinguishes local repeatability, transfer of the same procedure to another laboratory, and recovery of the same measurand by an independent measurement route (or technique).

Comparability also requires traceability of important inputs. In the progress-curve example, the molar absorption coefficient establishes the concentration scale and the active-site measurement establishes the scale for *k*_cat_. If those inputs are untraceable or incompletely characterized, repetition of the kinetic run cannot reveal the resulting common bias.^1,2,22^

## Existing Standards and the Contribution of This Perspective

The measurement-science foundation already exists. The VIM establishes the vocabulary of the measurand, measurement result, and traceability. The GUM makes uncertainty part of the reported result, while GUM-6 explains how measurement models are developed and used. IUPAC and Eurachem translate those principles into chemical practice.^1–3,5,22^

Biochemistry has complementary infrastructure. The Standards for Reporting Enzymology Data, known as STRENDA, specify the *minimum* information that must accompany an enzyme-kinetic measurement for it to be interpretable, and STRENDA DB validates structured submissions against those requirements.^6,7,23^ EnzymeML preserves reaction conditions and time courses in a machine-readable record, together with the associated models and fitted parameters, while the System for the Analysis of Biochemical Pathways–Reaction Kinetics (SABIO-RK) curates kinetic results and PEtab encodes parameter-estimation problems for systems biology.^12,13,24,25^

These efforts address different parts of the measurement. General metrology does not by itself determine whether a particular biochemical approximation is valid or whether a progress curve constrains *K*_M_. STRENDA improves experimental reporting, yet a complete STRENDA record need not contain an identifiability analysis or a full uncertainty budget for a model-derived parameter. EnzymeML and PEtab make data and models portable, but portability alone does not establish that the intended parameter was measured.

The contribution of this Perspective is the linking architecture. It begins with a biochemical measurand and follows the complete chain from material and signal to model, inference, and uncertainty. It requires evidence both that the biochemical reduction is valid and that the experimental design constrains the intended parameter. It then places local repetition, transfer across laboratories, and agreement across independent routes (or techniques) within the same evidential framework. The prioritized recommendations later in the paper show how these requirements can be adopted without treating every routine assay as a reference measurement.

Clinical enzymology demonstrates that such integration is feasible. The International Federation of Clinical Chemistry and Laboratory Medicine has developed primary reference procedures for catalytic activities at 37 °C. These procedures specify the measurand and the measurement equation, and document both the uncertainty budget and the calibration hierarchy.^26^ The same discipline can be extended beyond catalytic activity to kinetic and binding constants. The infrastructure required should remain proportional to the claim.

## Ligand–Receptor Binding as a Second Case

Consider reversible one-site binding,

10 $$R+L\underset{k_{\mathrm{off}}}{\overset{k_{\mathrm{on}}}{⇌}}\mathrm{RL},,K_{D}=\frac{k_{\mathrm{off}}}{k_{\mathrm{on}}}=\frac{\left[R\right]\left[L\right]}{\left[\mathrm{RL}\right]}\mathrm{at equilibrium}$$

The reported dissociation constant depends on which free species and binding-competent material have been defined, on whether equilibrium was reached, and on how the bound complex generated the recorded signal. A label or immobilized surface can therefore make the fitted quantity method-specific even when the curve is smooth and repeatable.^27,28^

The depletion-aware binding equation follows from mass balance. Writing *c* = [RL], so that [R] = [R]_T_ – *c* and [L] = [L]_T_ – *c*, substitution into eq 10 yields a quadratic in *c* whose physically admissible solution is the smaller root,

11 $$\left[\mathrm{RL}\right]=\frac{1}{2}\left\{{[R]}_{T}+{[L]}_{T}+K_{D}-\sqrt{{\left({\left[R\right]}_{T}+{\left[L\right]}_{T}+K_{D}\right)}^{2}-4{[R]}_{T}{[L]}_{T}}\right\}$$

because it satisfies 0 ≤ [RL] ≤ min­{[R]_T_, [L]_T_}.

A fluorescence experiment still requires an observation equation, for example

12 $$Y_{i}=Y_{0}+\Delta Y\frac{{[\mathrm{RL}]}_{i}}{{[L]}_{T,i}}+\varepsilon_{i}$$

when the signal reports the fraction of labeled ligand bound. Here *Y*_0_ is the unbound baseline and Δ*Y* is the response amplitude. Equation 12 becomes part of a statistical measurement model once the variation in *ε*_*i*_ is specified.^3,27,28^

In the illustration that follows, the labeled ligand is held at a fixed total concentration and the receptor is titrated, as in a fluorescence-anisotropy assay. Figure 4 shows two consequences. When total labeled ligand is not negligible, binding depletes free titrant and displaces the midpoint of the observed curve to

13 $${[R]}_{T,1/2}=K_{D}+\frac{1}{2}{[L]}_{T}$$

so that a midpoint-based or simple-hyperbolic interpretation, which ignores mass balance, assigns an apparent half-saturation constant displaced by half the tracer concentration (Figure 4A). The displacement of the midpoint is exact; what an arbitrary least-squares hyperbola returns for a curve that is not a hyperbola depends on the fitted range and weighting. The information about *K*_D_ is also concentrated near the transition region. Low-concentration observations confound *K*_D_ with the unknown amplitude, whereas saturated observations mainly define the plateau (Figure 4B).

**Figure 4:**
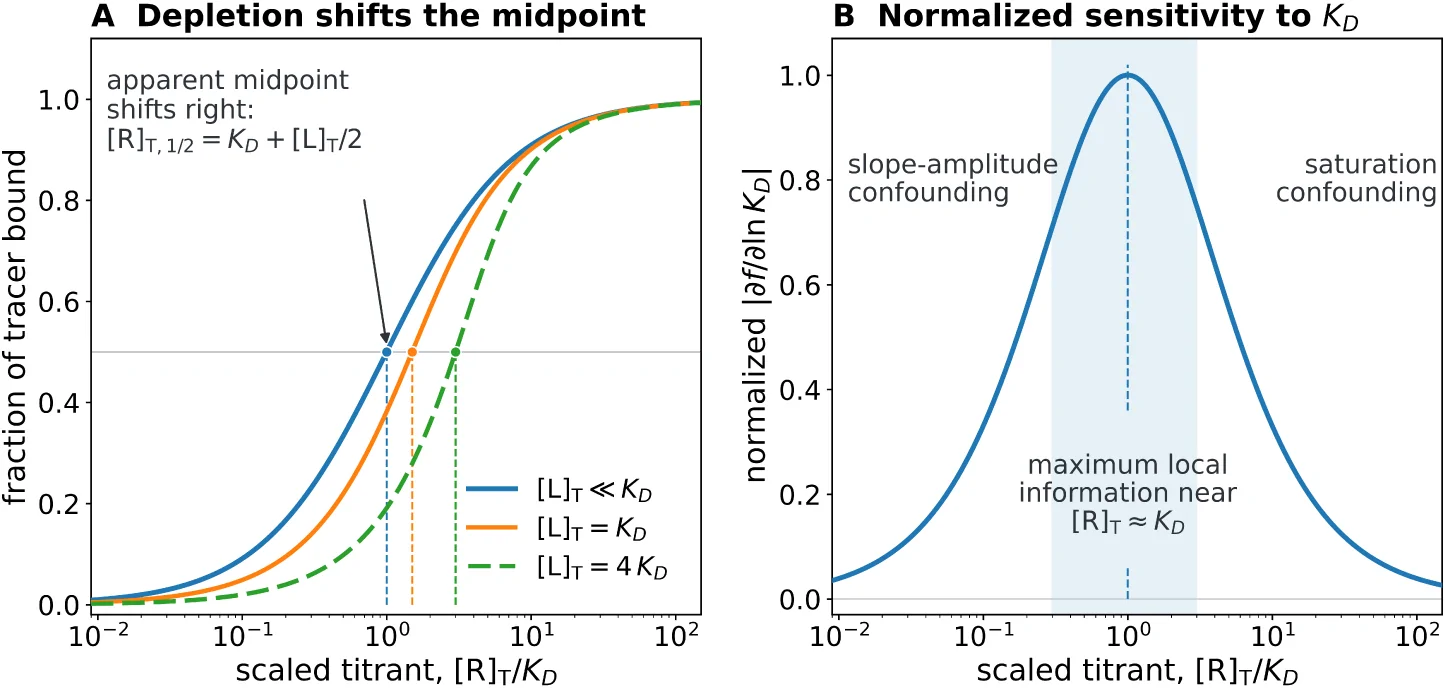
Ligand–receptor binding illustrates depletion and parameter confounding. **A** Increasing the total labeled-ligand concentration displaces the midpoint of the binding curve to [R]_T,1/2_, even though the curve remains smooth and sigmoidal on a logarithmic titrant scale; the curves plotted are the exact mass-balance solution, which is not a hyperbola in [R]_T_. **B** For the low-depletion limiting curve *f* = *u*/(1 + *u*), with *u* = [R]_T_/*K*_D_, the panel shows the normalized magnitude 4|*∂f*/*∂* ln *K*_D_|; the factor of four makes its maximum equal to one. Information about *K*_D_ is greatest near the transition region, whereas observations far below or above it chiefly determine a parameter combination or the response plateau.

Kinetic binding presents the same separation problem. Under a pseudo-first-order approximation the observed rate constant is *k*_obs_ = *k*_on_[L] + *k*_off_, so association experiments at several ligand concentrations are needed to separate *k*_on_ from *k*_off_. A single exponential at one concentration can fit well while the microscopic constants remain weakly constrained.^28,29^

A complete binding report would first identify the free receptor and ligand constructs and establish the binding-competent concentrations under stated chemical conditions. It would then give the mass-balance equation, the observation equation, and the error model. The concentration range should show that the transition was observed. The report should give an interval for *K*_D_, account for uncertainty from concentration and calibration, and state how labeling or immobilization might alter the result, treating depletion and linked equilibria explicitly where they are present.

## Does the Experiment Constrain the Parameter?

The form of variation is part of the measurement model. Constant-variance error may be reasonable on a raw absorbance scale and inappropriate after a nonlinear signal transformation. Drift, timing error, or repeated measurements from the same preparation can also create correlation. The estimator and its interval therefore cannot be interpreted apart from the observation scale and error structure.^2,22,30^

Whichever likelihood *L*(*θ*) is adopted, the assumed variance structure enters it alongside the model-predicted mean, so the estimator and its interval inherit that assumption; the Supporting Information gives the form used for the worked exemplar.

Identifiability asks whether the specified experiment could have produced useful bounds on the parameter being reported. Structural identifiability addresses ideal, noise-free observations of the model. Practical identifiability addresses the experiment actually performed. Its assessment depends on the concentration range and sampling schedule, as well as on the noise and calibration. A parameter can satisfy the first condition and remain unbounded in a particular experiment.^8–10,31^

A profile likelihood provides an operational check. One parameter is fixed at a sequence of values, the remaining parameters are re-estimated, and the best conditional fits are compared with the overall best fit. Crossing the chosen threshold on both sides gives a finite interval; failure to cross in one direction shows that the data do not place a finite bound there. Sensitivity analysis identifies the observations that carry information, while simulation-and-recovery tests whether the proposed design can recover known values under realistic variation.^8,9,32^

Type A and Type B evaluations address the sources that contribute after the target has been identified. A standard uncertainty derived from a fitted covariance is a Type A contribution; calibration, material characterization, and reference information contribute Type B components. A profile-likelihood region is a different object. It is an interval diagnostic of what the data constrain, not a standard uncertainty, and it should not be combined with Type B inputs by root-sum-of-squares. Where the likelihood is markedly asymmetric the two will disagree, and the profile region is the more faithful account of what the experiment supports. The combined uncertainty must reflect the inputs relevant to the declared measurand. When local propagation is unreliable, GUM Supplement 1 provides Monte Carlo propagation through the measurement model.^2,22,33^

Experimental design should follow the inverse question. Enzyme progress curves should sample the region around *K*_M_ and characterize active enzyme separately. Binding experiments should cover the transition in the binding curve and quantify the binding-competent material. Replication is most useful when allocated to the sources that dominate the final uncertainty. The corresponding local sensitivity and Fisher-information equations are provided in the Supporting Information.^21,28^

## From Repeatability to Interlaboratory Evidence

Terminology differs across communities, so the evidential distinctions should be stated explicitly. Repeatability asks whether the same team and apparatus produce compatible results under closely matched conditions. Transfer replication asks whether a specified procedure can be implemented in another competent laboratory. Independent-method reproducibility asks whether a different physical route (or technique) recovers the same measurand within uncertainty; in the notation of eq 7–9 it asks whether *q̂*_1_ and *q̂*_2_ are compatible when *g*_*m*_, *η*_*m*_, and *C*_*m*_ all differ, and it is therefore the strongest available test of the invariance of *q* to the route (or technique) by which it was obtained. ISO intermediate precision, which varies days, operators, or calibrations within one laboratory, lies between the first two.^1,34,35^ Table 3 states what each check establishes and, equally important, what it cannot establish on its own.

**Table 3:** Three Evidential Checks on a Biochemical Measurement

Check	What it establishes	What it cannot establish alone
Repeatability	Local execution and short-term precision under closely matched operator, material, instrument, and calibration conditions	Whether the procedure reaches the intended measurand, or whether it carries a stable bias
Transfer replication	Completeness of the written procedure and its robustness to a change of laboratory, operator, and ordinary materials	Whether a shared model assumption or method-specific artifact biases every laboratory alike
Independent-method reproducibility	Whether the specified measurand survives a change of observable, calibration chain, nuisance structure, and often model	Whether every perturbing mechanism has been excluded, or which route (or technique) is closer to the true value when they disagree

An interlaboratory study with a common material and procedure tests whether the method travels and whether the written record contains the knowledge needed to use it. Hierarchical designs can distinguish laboratory-level and material-level variation, including contributions from calibration and analysis. The observed spread can also be compared with the participants’ uncertainty budgets. Dispersion that remains unexplained is often called *dark uncertainty*; it signals a missing or underestimated source that should be investigated.^36^

The design of the comparison determines the scientific question. Holding the procedure fixed tests transfer. Deliberately changing the measurement route (or technique) tests whether the measurand is stable to different observables and nuisance structures. The Yeast Systems Biology Network and STRENDA study illustrates the first role by comparing enzyme activities under standardized, in-vivo-like conditions.^37^

Independent routes (or techniques) provide a stronger test when their dominant errors differ. For example, a binding result obtained by calorimetry can be compared with a solution-phase optical method or a surface method, provided shared concentration standards and other common inputs are audited. Agreement then argues that the result survives changes in signal generation and analysis. Disagreement is also informative, indicating that the measurand was under-specified, that the material differed, or that an approximation was invalid or a design uninformative.^27,28,38,39^

## Prioritized Recommendations for Routine Analytical Biochemistry

The evidential burden should rise with the claim. A screening quantity used to rank compounds does not require the same support as a constant interpreted within a specified molecular reaction mechanism or intended for reference use. Table 4 separates three levels of practice.

**Table 4:** Prioritized Implementation of Measurand-Centered Practice

Level	Evidence to provide	Appropriate use
Minimum—every quantitative study	Declare the measurand and conditions, preserve the signal and calibration, state the models and estimator, and report an interval with basic diagnostics and software information	Routine quantitative results and operational comparisons
Recommended—defensible parameter estimation	Establish the active or binding-competent material, test the validity regime, examine parameter bounds, and include important non-fit uncertainty contributions. Use recovery or robustness checks where the design is delicate	Parameters used for mechanism-based interpretation, modeling, comparison, or reuse
Reference-grade—high-confidence constants	Add full traceability, an independent measurement route (or technique), interlaboratory evidence, and explicit assessment of model-form and long-term stability	Reference quantities, standards, and strong claims about molecular mechanism

The minimum level can be incorporated into an ordinary paper with a short measurand statement and a transparent analysis paragraph. The recommended level adds evidence that the chosen model and experimental range support the parameter. Reference-grade work requires infrastructure that most individual laboratories cannot supply alone, including comparison materials and coordinated studies.

Adoption has real costs. Some laboratories lack metrological or statistical expertise, and active or binding-competent concentrations may be difficult to establish. Independent routes (or techniques) and interlaboratory studies also require resources. Proportional implementation keeps the framework practical: every study can declare its target, preserve the measured signal, state the model, and carry the relevant uncertainty into the reported result, with stronger evidence added as the claim warrants it.

Figure 5 shows what the recommended level looks like when written out for the worked case study. It is the concrete form of Table 2 and is short enough to sit in an ordinary methods section.

**Figure 5:**
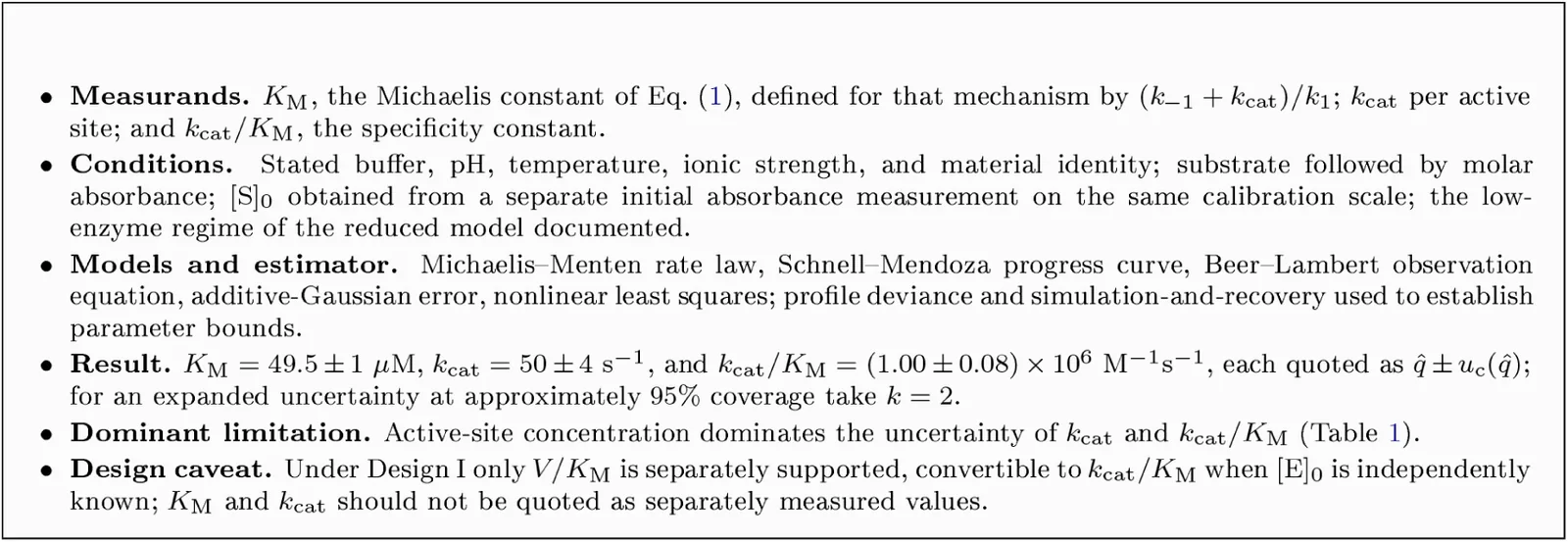
Measurand declaration for Design II of the Michaelis–Menten progress-curve example.

Software can lower the entry barrier. PyEnzyme provides a high-level interface to the EnzymeML data model, and LMFIT offers accessible nonlinear estimation and confidence-interval tools within the Scientific Python ecosystem.^40,41^ The inferential convention still has to be reported, since packages differ in how they encode likelihoods and weighting, in the bounds they impose, and in the way they construct intervals. The explicit code for the worked example is supplied as a transparent reference implementation rather than an expectation that each laboratory reproduce the analysis from first principles.

A portable record makes that evidence reusable, and the FAIR principles—that data be findable, accessible, interoperable, and reusable—set the standard it should meet.^11^ Such a record should preserve the assay description together with the raw signal and calibration, and the executable models and parameter-estimation specification should travel with the diagnostics and the uncertainty budget. A persistent identifier and a record of the software environment then connect those objects to the reported measurand; deposition in an archive that mints such an identifier, rather than in a mutable code repository alone, is what makes the link durable. EnzymeML and PEtab already encode important parts of this structure.^12,13,25^

## Discussion and Outlook

The recommendations in this Perspective are intended to connect existing standards rather than replace them. IUPAC, the VIM, and the GUM provide the general grammar of measurement and uncertainty. STRENDA specifies the experimental information needed to interpret enzyme-kinetic data. EnzymeML and PEtab make data, models, and estimation problems exchangeable, while SABIO-RK supports curation and reuse. Each contribution is necessary, but each addresses a different layer of the measurement.

The additional step developed here is evaluative. A biochemical constant is followed from the material and signal to its model-defined target. Before that parameter is reported, the biochemical reduction and the information supplied by the design are both examined. Uncertainty from calibration and material characterization is then combined with fit uncertainty. Finally, the claim is tested at an appropriate level: local repeatability, laboratory transfer, or agreement across independent routes (or techniques). This architecture turns terminology, reporting, and data exchange into a single signal-to-measurand workflow.

The Michaelis–Menten example shows why that connection matters. A valid reduced equation and a close fit to the measured signal do not ensure that *K*_M_ was separately determined, and a precise fit does not account for uncertainty in active enzyme. Ligand–receptor binding shows the same dependence on mass balance, observation equations, and experimental range. These are general features of analytical measurements that infer biochemical quantities from signals.

The agenda that follows is practical rather than aspirational: the minimum level of Table 4 can be adopted immediately by any laboratory, and interlaboratory evidence and independent measurement routes (or techniques) are added as the consequence of the claim demands. Reproducibility is achieved when the meaning, information content, and uncertainty of the biochemical measurand remain explicit across that entire chain.

## Supplementary Material



## Data Availability

No new experimental data were generated for this Perspective. The fixed-seed synthetic observations used in the worked exemplar are deposited with the analysis code. Running run_all.py regenerates all manuscript figures and the values in Supporting Information Tables S1–S3 and S5 using seed 20240530; code/validate_schedules.py separately regenerates Table S4 using seed 20240531. The code is openly available at https://github.com/santiago-schnell/signals-to-measurands. All other claims rest on previously published literature listed in the References.

## Notes

Complete signal-to-measurand record for the worked Michaelis–Menten exemplar; measurement and observation models; likelihood and profile-deviance definitions; simulation-and-recovery and sampling-schedule studies; uncertainty propagation; computational diagnostics (PDF)
