# Supplementary material for: From Signals to Measurands: A Measurement-Science Roadmap for Reproducible Analytical Biochemistry
Source: ACS Meas Sci Au. 2026 Sep 7;6(5):1365–74. doi: 10.1021/acsmeasuresciau.6c00198 (PMC13693181; doi:10.1021/acsmeasuresciau.6c00198)
Supplement: Supplementary file 1 [file tg6c00198_si_001.pdf]

Supporting Information

From Signals to Measurands: A Measurement-Science Roadmap for Reproducible Analytical Biochemistry

Santiago Schnell

Department of Mathematics, Dartmouth, Hanover, NH 03755, USA

Departments of Biochemistry & Cell Biology and Biomedical Data Science,

Geisel School of Medicine at Dartmouth, Hanover, NH 03755, USA

This document provides the complete signal-to-measurand record for the worked Michaelis–Menten progress-curve exemplar in the main text. It specifies the measurement and observation models, estimator, profile-deviance analysis, simulation-and-recovery study, sampling-schedule comparison, uncertainty propagation, and computational record. The data are simulated; the purpose is to demonstrate an auditable inferential workflow rather than to characterize a particular enzyme.

S1 Scenario and ground truth

Substrate consumption is followed spectrophotometrically. The Beer–Lambert measurement equation is

$$A = \varepsilon\,\ell\,[\mathrm{S}], \tag{S1}$$

where A is absorbance, ε is the molar absorption coefficient, ℓ is the optical path length, and [S] is the free-substrate concentration. Thus, ε and ℓ establish the concentration scale used throughout the analysis. The initial substrate concentration is a separately calibrated input obtained from an independent initial absorbance measurement, $[\mathrm{S}]_0 = A_{0,\mathrm{cal}}/(\varepsilon\ell)$, on that same scale. It is not inferred from the first noisy observation in the progress curve. Repeatability uncertainty in $A_{0,\mathrm{cal}}$ and uncertainty in the 1 cm path length are neglected in this illustrative budget.

Two substrate-depletion experimental designs are evaluated under the same biochemical parameters and absorbance-noise model. Design I starts at $[\mathrm{S}]_0 = 0.2K_\mathrm{M}$ and remains in the low-substrate regime, $[\mathrm{S}] \ll K_\mathrm{M}$. Design II starts at $[\mathrm{S}]_0 = 4K_\mathrm{M}$ and traverses concentrations on the order of K_M. The nominal total enzyme concentration is denoted $[\mathrm{E}]_\mathrm{tot}$; ϕ is the fraction of catalytically active sites; and

$$[\mathrm{E}]_0 = \phi[\mathrm{E}]_\mathrm{tot} \tag{S2}$$

is the initial active-site concentration used to define the turnover number. This convention is used throughout the main text and Supporting Information. Ground-truth and instrument values are listed in Table S1.

S2 Measurement equation and observation model

The biochemical model is the reduced Michaelis–Menten rate law

$$\frac{\mathrm{d}[\mathrm{S}]}{\mathrm{d}t} = -\frac{V[\mathrm{S}]}{K_\mathrm{M} + [\mathrm{S}]}, \qquad V = k_\mathrm{cat}[\mathrm{E}]_0, \tag{S3}$$

Table S1: Ground-truth biochemical parameters and instrument model. AU, absorbance unit.

Quantity	Symbol	Value
Michaelis constant	K_M	50 μM
Turnover number	k_cat	50 s^{-1}
Nominal total enzyme concentration	$[\mathrm{E}]_\mathrm{tot}$	5.0 nM
Active fraction	ϕ	0.80
Initial active-site concentration	$[\mathrm{E}]_0 = \phi[\mathrm{E}]_\mathrm{tot}$	4.0 nM
Limiting rate	$V = k_\mathrm{cat}[\mathrm{E}]_0$	0.20 $\mu\mathrm{M\,s}^{-1}$
Specificity constant	$k_\mathrm{cat}/K_\mathrm{M}$	$1.00 \times 10^6\ \mathrm{M}^{-1}\mathrm{s}^{-1}$
Molar absorption coefficient	ε	6220 $\mathrm{M}^{-1}\mathrm{cm}^{-1}$
Path length	ℓ	1.0 cm
Absorbance-noise standard deviation	σ_A	0.003 AU
Concentration-noise standard deviation	$\sigma_S = \sigma_A/(\varepsilon\ell)$	0.48 μM

where t is time, V is the limiting rate, and K_M is the Michaelis constant. For initial condition $[\mathrm{S}](0) = [\mathrm{S}]_0$, its closed-form progress curve is

$$[\mathrm{S}](t; V, K_\mathrm{M}, [\mathrm{S}]_0) = K_\mathrm{M} W_0\left[\frac{[\mathrm{S}]_0}{K_\mathrm{M}} \exp\left(\frac{[\mathrm{S}]_0 - Vt}{K_\mathrm{M}}\right)\right], \tag{S4}$$

where W_0 is the principal real branch of the Lambert W function. Equation (S4) is the Schnell–Mendoza equation.[1] Goudar and colleagues illustrated its effectiveness for progress-curve fitting.[2]

The observation model places additive, independent Gaussian noise on absorbance:

$$A_\mathrm{obs}(t_i) = \varepsilon\ell\,[\mathrm{S}](t_i; V, K_\mathrm{M}, [\mathrm{S}]_0) + \eta_i, \qquad \eta_i \overset{\mathrm{iid}}{\sim} \mathcal{N}(0, \sigma_A^2), \quad i = 1, \ldots, n, \tag{S5}$$

where $A_\mathrm{obs}(t_i)$ is the observed absorbance at sampling time t_i, n is the number of observations, η_i is the absorbance error, and σ_A is its standard deviation; "iid" means independently and identically distributed. The concentration trace used for fitting is

$$[\mathrm{S}]_\mathrm{obs}(t_i) = \frac{A_\mathrm{obs}(t_i)}{\varepsilon\ell}, \qquad \sigma_S = \frac{\sigma_A}{\varepsilon\ell}. \tag{S6}$$

Under Eq. (S5), $[\mathrm{S}]_\mathrm{obs}(t_i)$ has a constant standard deviation σ_S. Because the Gaussian error is placed on absorbance, transformed concentration estimates may be nonpositive when the residual signal is smaller than the baseline noise. The three such Design I observations are retained at their observed values in the fit; Figure 1A displays them at a declared plotting floor because its ordinate is logarithmic.

S3 Estimator, likelihood, and profile deviance

The parameter vector $\boldsymbol{\theta} = (V, K_\mathrm{M})^\mathsf{T}$ is estimated by nonlinear least squares (NLS) on the concentration trace. Because V and K_M have different units and poorly matched numerical scales in the chosen parameterization, the numerical fit uses $\log V$ and $\log K_\mathrm{M}$ and is repeated from five starting points. Under Eq. (S5), NLS is equivalent to maximizing the Gaussian log-likelihood

$$\log L(V, K_\mathrm{M}) = -\frac{1}{2\sigma_S^2} \sum_{i=1}^{n} \left[[\mathrm{S}]_\mathrm{obs}(t_i) - [\mathrm{S}](t_i; V, K_\mathrm{M}, [\mathrm{S}]_0)\right]^2 + \mathrm{const.} \tag{S7}$$

Define the residual sum of squares

$$\mathrm{SSR}(V, K_\mathrm{M}) = \sum_{i=1}^{n} \left[[\mathrm{S}]_\mathrm{obs}(t_i) - [\mathrm{S}](t_i; V, K_\mathrm{M}, [\mathrm{S}]_0) \right]^2, \tag{S8}$$

and let $\mathrm{SSR}^\star$ be the lowest residual sum of squares found by the stated bounded multistart procedure. This is the best-fit numerical reference used below; no analytical proof of global optimality is claimed. Practical identifiability is assessed by profiling the likelihood.[3,4] For each fixed value of K_M, V is re-estimated, giving the profile log-likelihood $\ell_p(K_\mathrm{M})$. The profile deviance is

$$D_p(K_\mathrm{M}) = -2\{\ell_p(K_\mathrm{M}) - \ell_\mathrm{max}\} \tag{S9}$$

$$= \frac{\mathrm{SSR}_\mathrm{min}(K_\mathrm{M}) - \mathrm{SSR}^\star}{\sigma_S^2}, \qquad \mathrm{SSR}_\mathrm{min}(K_\mathrm{M}) = \min_V \mathrm{SSR}(V, K_\mathrm{M}). \tag{S10}$$

Under the usual likelihood-ratio regularity conditions, $D_p(K_\mathrm{M})$ is compared with a χ_1^2 distribution; the nominal 95% profile region is $\{K_\mathrm{M} : D_p(K_\mathrm{M}) \le 3.841\}$. The combination $\psi = V/K_\mathrm{M}$ is profiled by imposing $V = \psi K_\mathrm{M}$ and minimizing over K_M.

For Design I, the absence of a finite upper bound is also checked at the limiting end of the profile. As $K_\mathrm{M} \to \infty$ with $\psi = V/K_\mathrm{M}$ fixed, Eq. (S3) approaches

$$[\mathrm{S}](t) = [\mathrm{S}]_0 \exp(-\psi t). \tag{S11}$$

Re-estimating ψ in Eq. (S11) gives $D_p(\infty) = 1.6773 < 3.841$. The nominal 95% profile region therefore remains open as $K_\mathrm{M} \to \infty$; this conclusion does not depend on the finite 50 mM plotting limit.

The reduced chi-square reported below is

$$\chi^2_\mathrm{red} = \frac{\mathrm{SSR}^\star}{(n-p)\sigma_S^2}, \qquad p = 2, \tag{S12}$$

where p is the number of fitted parameters. Results are summarized in Table S2. SE denotes standard error and CI denotes confidence interval. Both designs satisfy forward adequacy, but only Design II yields a finite upper profile-deviance bound for K_M. Design I principally constrains the combination V/K_M, as expected from its low-substrate trajectory.[5]

Table S2: Fit quality, point estimates, Type A standard errors, and profile-deviance confidence intervals. "$\uparrow \infty$" denotes no finite upper 95% bound.

	**Design I** ($[\mathrm{S}]_0 = 0.2K_\mathrm{M}$)	**Design II** ($[\mathrm{S}]_0 = 4K_\mathrm{M}$)
Reduced χ^2	1.02	1.13
$\hat{V}$ ($\mu\mathrm{M\,s^{-1}}$)	0.167	0.200
Type A SE on V	77%	0.4%
$\widehat{K_\mathrm{M}}$ (μM)	39.5	49.5
Type A SE on K_M	88%	1.3%
K_M 95% profile CI (μM)	[12.8, $\uparrow \infty$)	[48.4, 50.5]
V/K_M 95% profile CI ($10^{-3}\,\mathrm{s^{-1}}$)	[3.6, 5.3]	[3.97, 4.11]

S4 Local sensitivity and Fisher information

The main text uses plain-language identifiability diagnostics. For completeness, the corresponding local calculation is recorded here. Let

$$\widehat{S}_i(\boldsymbol{\theta}) = [\mathrm{S}](t_i; V, K_\mathrm{M}, [\mathrm{S}]_0), \qquad \boldsymbol{\theta} = (V, K_\mathrm{M})^\mathsf{T}, \tag{S13}$$

and define the sensitivity vector

$$\mathbf{s}_i(\boldsymbol{\theta}) = \frac{\partial \widehat{S}_i(\boldsymbol{\theta})}{\partial \boldsymbol{\theta}}. \tag{S14}$$

For the homoscedastic Gaussian observation model in Eq. (S5), the mean-response contribution to the Fisher information is

$$\mathcal{I}(\boldsymbol{\theta}) = \frac{1}{\sigma_S^2} \sum_{i=1}^{n} \mathbf{s}_i(\boldsymbol{\theta}) \mathbf{s}_i(\boldsymbol{\theta})^\mathsf{T}. \tag{S15}$$

When the sensitivity vectors are nearly collinear over the sampling schedule, $\mathcal{I}$ is nearly singular and only a parameter combination is locally constrained. In the low-substrate limit, $\mathrm{d}[\mathrm{S}]/\mathrm{d}t \simeq -(V/K_\mathrm{M})[\mathrm{S}]$, so changes in V and K_M that preserve V/K_M have nearly the same effect on the curve. This is the local counterpart of the unbounded profile in Design I.

Under standard regularity conditions, the inverse information matrix provides a local covariance approximation and enters the Cramér–Rao bound for unbiased estimators. These local results are useful design diagnostics, but they do not replace the profile deviance or simulation-and-recovery when the likelihood is asymmetric, the optimum is near a boundary, or the information matrix is nearly singular.[3,4,6]

S5 Simulation-and-recovery

For each design, 400 synthetic data sets were generated at the ground-truth parameters and fitted using the same schedule, bounds, and estimator as the worked data set. Table S3 reports the median and central 95% of recovered estimates. In Design I, recovered K_M values span nearly three orders of magnitude and 16% of fits reach the upper search bound of 10 mM; in Design II, K_M and V/K_M recover tightly. The study therefore distinguishes a local fit that looks adequate from a design that reliably recovers the declared parameters.

Table S3: Simulation-and-recovery over 400 synthetic data sets per design: median and central 95% of recovered estimates.

	**Design I**	**Design II**
K_M median (μM)	55	50.0
K_M central 95% (μM)	[15, 1.0×10^4]	[48.6, 51.3]
Fraction of fits at the 10 mM cap	16% (65/400)	0%
V/K_M median ($10^{-3}\,\mathrm{s}^{-1}$)	3.97	4.00
V/K_M central 95% ($10^{-3}\,\mathrm{s}^{-1}$)	[3.49, 4.98]	[3.93, 4.08]

S6 Sampling-schedule comparison

Designs I and II differ not only in $[\mathrm{S}]_0$ but also in the observation window and the number of observations, because the two designs deplete substrate on very different timescales: Design I is

sampled at 50 points over 1000 s and Design II at 60 points over 1800 s. The comparison in Table S4 establishes that the non-identifiability of K_M under Design I is a property of the substrate regime rather than of sampling effort.

For each row, 100 synthetic data sets were generated under the indicated design and sampling schedule, and K_M was profiled over the interval [1 μM, 10 mM]. Two summaries are reported: the proportion of replicates whose nominal 95% profile region reaches the upper end of that interval without closing, and the median width of the region on a logarithmic scale. Giving Design I the schedule of Design II, or four times as many observations, or five times the observation window, leaves the region spanning between two and three orders of magnitude. Design II closes the region in every replicate.

Table S4: Sampling-schedule comparison over 100 synthetic data sets per row, generated under the indicated design and schedule. Width is the median span of the nominal 95% profile region for K_M, in decades; the profiling grid used for this comparison resolves widths to one grid step, 0.11 decades.

**Schedule**	**Region open at 10 mM limit**	**Median width**
Design I, as reported ($n = 50$, 1000 s)	84%	2.74
Design I, Design II schedule ($n = 60$, 1800 s)	91%	2.80
Design I, $n = 200$ over 1800 s	63%	2.40
Design I, $n = 300$ over 5000 s	76%	2.63
Design II, as reported ($n = 60$, 1800 s)	0%	< 0.11

For the noise level and sampling efforts considered here, adding observations within the low-substrate regime does not make K_M recoverable. Changing the substrate regime is substantially more effective than collecting additional observations within that regime.[5]

S7 Uncertainty budget and propagation

The uncertainty budget is constructed for Design II, where K_M is identified. In the Guide to the Expression of Uncertainty in Measurement (GUM), a Type A component is evaluated statistically from measured or fitted data, whereas a Type B component is evaluated from other information.[7] Here, the Type A component is the standard uncertainty obtained from the fitted covariance of (V, K_M). The profile-deviance regions reported above serve a different purpose: they are interval diagnostics of identifiability, and are not standard uncertainties that may be combined with Type B inputs by root-sum-of-squares. Where the likelihood is markedly asymmetric the two will disagree, and the profile region is then the more faithful description of what the data support. The two Type B inputs are the molar absorption coefficient, $u(\varepsilon)/\varepsilon = 2\%$, and the initial active-site concentration, $u([\mathrm{E}]_0)/[\mathrm{E}]_0 = 8\%$, established by active-site titration.

Let $\boldsymbol{\Sigma}$ be the fitted covariance matrix of (V, K_M). The Type A variance of $\psi = V/K_\mathrm{M}$ follows from the delta method:

$$u_\mathrm{A}^2(\psi) = \mathbf{g}^\mathsf{T}\boldsymbol{\Sigma}\mathbf{g}, \qquad \mathbf{g} = \left(\frac{1}{K_\mathrm{M}}, -\frac{V}{K_\mathrm{M}^2}\right)^\mathsf{T}, \tag{S16}$$

where $\mathbf{g} = \partial(V/K_\mathrm{M})/\partial(V, K_\mathrm{M})$ is the gradient evaluated at the estimate. The notation $u_\mathrm{A}(X)$ denotes a Type A standard uncertainty in X, and $u_\mathrm{c}(X)$ denotes its combined standard uncertainty.

Every concentration in this exemplar is defined on one absorbance scale by division by $\varepsilon\ell$. The progress-curve observations and the separately calibrated input $[\mathrm{S}]_0 = A_{0,\mathrm{cal}}/(\varepsilon\ell)$ therefore covary when that scale changes. A relative change in ε rescales both by the same inverse factor, so that $V \propto \varepsilon^{-1}$ and $K_\mathrm{M} \propto \varepsilon^{-1}$ and their ratio V/K_M is invariant to ε. Were $[\mathrm{S}]_0$ instead assigned independently

of this scale, the cancellation would fail and its uncertainty and covariance with ε would have to be carried explicitly. The analysis code verifies the stated convention numerically by refitting under a perturbed ε, recovering $\partial \ln V/\partial \ln \varepsilon = \partial \ln K_\mathrm{M}/\partial \ln \varepsilon = -1.0000$ and $\partial \ln(V/K_\mathrm{M})/\partial \ln \varepsilon = 0.0000$. Using $k_\mathrm{cat} = V/[\mathrm{E}]_0$ and $k_\mathrm{cat}/K_\mathrm{M} = (V/K_\mathrm{M})/[\mathrm{E}]_0$, and treating the Type A fit, ε, and $[\mathrm{E}]_0$ inputs as independent, the relative combined standard uncertainties are

$$\left[\frac{u_\mathrm{c}(K_\mathrm{M})}{K_\mathrm{M}}\right]^2 = \left[\frac{u_\mathrm{A}(K_\mathrm{M})}{K_\mathrm{M}}\right]^2 + \left[\frac{u(\varepsilon)}{\varepsilon}\right]^2, \tag{S17}$$

$$\left[\frac{u_\mathrm{c}(k_\mathrm{cat})}{k_\mathrm{cat}}\right]^2 = \left[\frac{u_\mathrm{A}(V)}{V}\right]^2 + \left[\frac{u(\varepsilon)}{\varepsilon}\right]^2 + \left[\frac{u([\mathrm{E}]_0)}{[\mathrm{E}]_0}\right]^2, \tag{S18}$$

$$\left[\frac{u_\mathrm{c}(k_\mathrm{cat}/K_\mathrm{M})}{k_\mathrm{cat}/K_\mathrm{M}}\right]^2 = \left[\frac{u_\mathrm{A}(V/K_\mathrm{M})}{V/K_\mathrm{M}}\right]^2 + \left[\frac{u([\mathrm{E}]_0)}{[\mathrm{E}]_0}\right]^2. \tag{S19}$$

If input quantities are correlated, the corresponding covariance terms must be added rather than using these root-sum-of-squares expressions.[7,8]

Numerical values are collected in Table S5. The curve-fit standard error on k_cat (0.4%) is not the uncertainty of the final measurand: after the calibration and active-site inputs are included, the combined standard uncertainty is 8.3%. Rounded compatibility statements are

$$\frac{k_\mathrm{cat}}{K_\mathrm{M}} = (1.00 \pm 0.08) \times 10^6\ \mathrm{M^{-1}s^{-1}}, \quad K_\mathrm{M} = 49.5 \pm 1\ \mu\mathrm{M}, \quad k_\mathrm{cat} = 50 \pm 4\ \mathrm{s^{-1}}, \tag{S20}$$

where each entry is quoted as $\hat{q} \pm u_\mathrm{c}(\hat{q})$, a combined standard uncertainty. An expanded uncertainty would be $U = k\,u_\mathrm{c}$; for an approximately normal distribution and a coverage probability of about 95%, $k = 2$, so the corresponding expanded uncertainties are roughly twice the values shown. For nonlinear problems in which local Gaussian propagation is inadequate, the input distributions should instead be propagated through the measurement model by Monte Carlo simulation.[9]

Table S5: Relative standard uncertainties for the three Design II measurands.

**Measurand**	**Type A**	$u(\varepsilon)/\varepsilon$	$u([\mathrm{E}]_0)/[\mathrm{E}]_0$	u_c	**Dominant source**
K_M	1.3%	2.0%	—	2.4%	calibration scale (ε)
k_cat	0.4%	2.0%	8.0%	8.3%	active-site count ($[\mathrm{E}]_0$)
$k_\mathrm{cat}/K_\mathrm{M}$	0.9%	—	8.0%	8.1%	active-site count; ε cancels

S8 Code and data availability

The repository collectively reproduces the numerical results in Tables S1–S5. Running `run_all.py` regenerates all manuscript figures and the values in Tables S1, S2, S3, and S5 using seed 20240530. It also writes the fixed-seed synthetic observations to CSV files, `figures/numerical_summary.txt`, and the machine-readable result `figures/current_results.json`. The separate sampling-schedule study in Table S4 is regenerated separately by `code/validate_schedules.py` using seed 20240531; its result is written to `figures/schedule_validation.json`.

The record contains the synthetic signal and calibration constants; executable biochemical and observation models; the objective function, weighting, parameter bounds, and sampling designs; estimates, covariance matrices, profile deviances, local sensitivity and Fisher-information diagnostics, and simulation-and-recovery results; and the declared software environment and random

seeds. Tolerance-based tests compare newly generated results with immutable reference JSON files stored under `tests/`. The code is openly available at https://github.com/santiago-schnell/signals-to-measurands.

References

[1] Schnell, S.; Mendoza, C. Closed Form Solution for Time-Dependent Enzyme Kinetics. *J. Theor. Biol.* **1997**, *187*, 207–212, DOI: 10.1006/jtbi.1997.0425.

[2] Goudar, C. T.; Sonnad, J. R.; Duggleby, R. G. Parameter Estimation Using a Direct Solution of the Integrated Michaelis–Menten Equation. *Biochim. Biophys. Acta* **1999**, *1429*, 377–383, DOI: 10.1016/S0167-4838(98)00247-7.

[3] Raue, A.; Kreutz, C.; Maiwald, T.; Bachmann, J.; Schilling, M.; Klingmüller, U.; Timmer, J. Structural and Practical Identifiability Analysis of Partially Observed Dynamical Models by Exploiting the Profile Likelihood. *Bioinformatics* **2009**, *25*, 1923–1929, DOI: 10.1093/bioinformatics/btp358.

[4] Kreutz, C.; Raue, A.; Kaschek, D.; Timmer, J. Profile Likelihood in Systems Biology. *FEBS J.* **2013**, *280*, 2564–2571, DOI: 10.1111/febs.12276.

[5] Stroberg, W.; Schnell, S. On the Estimation Errors of K_M and V from Time-Course Experiments Using the Michaelis–Menten Equation. *Biophys. Chem.* **2016**, *219*, 17–27, DOI: 10.1016/j.bpc.2016.09.004.

[6] Tarantola, A. *Inverse Problem Theory and Methods for Model Parameter Estimation*; SIAM: Philadelphia, 2005.

[7] Evaluation of Measurement Data – Guide to the Expression of Uncertainty in Measurement, JCGM 100:2008; Joint Committee for Guides in Metrology, JCGM: 2008, DOI: 10.59161/JCGM100-2008E.

[8] Guide to the Expression of Uncertainty in Measurement – Part 6: Developing and Using Measurement Models, JCGM GUM-6:2020; Joint Committee for Guides in Metrology, JCGM: 2020, DOI: 10.59161/JCGMGUM-6-2020.

[9] Evaluation of Measurement Data – Supplement 1 to the Guide to the Expression of Uncertainty in Measurement – Propagation of Distributions Using a Monte Carlo Method, JCGM 101:2008; Joint Committee for Guides in Metrology, JCGM: 2008, DOI: 10.59161/JCGM101-2008.
